# Supplementary material for: Interventions to reduce sedentary behaviour in adults with type 2 diabetes: A systematic review and meta-analysis
Source: PLoS One. 2024 Jul 30;19(7):e0306439. doi: 10.1371/journal.pone.0306439 (PMC11288443; doi:10.1371/journal.pone.0306439)
Supplement: S1 Table — (DOCX) [file pone.0306439.s001.docx]

Table 1. Short-term SB interventions in adults with T2D

|  | **Study** | **Objective** | **Participants** | **Research Design** | **Intervention/ Comparison** | **Cardio Metabolic Outcomes** | **Main Results** | **RoB2**  **Quality** |
| --- | --- | --- | --- | --- | --- | --- | --- | --- |
| **1** | **(Blankenship et al., 2019)**  **[24]** | To investigate the effect of 20, 40, or 60 min of activity performed as either breaks from sitting after each meal (BR) or as one continuous walk after breakfast (WALK) on daily postprandial glycemia in adults with T2D. | N=30 (n=10/ condition 20, 40, or 60 min), mean age 64 ±8.2, 16 women [53.33%], mean BMI 31.7 | Randomized crossover design | Three 24-h experimental conditions in their free-living environment (with washout period): morning walk after breakfast (WALK), post-meal breaks from sitting (BR), and sedentary control (CON). Participants were randomized into the 20, 40, or 60 min of activity performed as either breaks from sitting after each meal (BR) or as one continuous walk after breakfast (WALK) daily. Standardized meals were consumed during each condition. To simulate real-world environments, participants chose the timing of the meals; however, participants were asked to replicate the time meals were consumed during each subsequent condition. Study conditions were monitored by activPAL. | The outcomes were postprandial glucose (PPG) across condition and the relationship between activity volume and glucose responses. A continuous glucose monitor (iPro2) was worn to quantify glycemic control. | BR and WALK significantly (P<.05) attenuated the breakfast PPG compared with CON. Lunch and dinner PPG were unaffected by BR and WALK.  BR and WALK did not significantly shorten the daily duration of hyperglycemia compared with CON (P>.05). | Some Concerns |
| **2** | **(Dempsey, Larsen, et al., 2016)**  **[25]** | To investigate the effect of interrupting prolonged sitting with brief bouts of light-intensity walking (LW) or simple resistance activities (SRA) on postprandial cardiometabolic risk markers in adults with T2D | N=24, mean age 62 ±6, 10 women [41.67%], mean BMI 33.0 | Randomized crossover design | Three 8h conditions on separate days (with washout period): uninterrupted sitting (control), sitting plus 3-min bouts of LW (3.2 km/h) every 30 min, and sitting plus 3-min bouts of SRA every 30 min. Standardized meals were consumed during each condition. Study conditions were monitored by accelerometer. | The outcomes were incremental areas under the curve (iAUCs) for glucose, insulin, c-peptide, and triglycerides. Blood was collected half-hourly. | The LW and SRA conditions produced significantly (P<.001) attenuated glucose, insulin, c-peptide, and triglyceride (SRA only) compared with sitting control. | Some Concerns |
|  | **(Dempsey, Sacre, et al., 2017)**  **[26]** | To investigate the effect of interrupting prolonged sitting with brief bouts of light-intensity walking (LW) or simple resistance activities (SRA) on blood pressure (BP) and plasma noradrenaline in adults with T2D | N=24, mean age 62 ±6, 10 women [41.67%], mean BMI 33.0 | Randomized crossover design | Three 8h conditions on separate days (with washout period): uninterrupted sitting (control), sitting plus 3-min bouts of LW (3.2 km/h) every 30 min, and sitting plus 3-min bouts of SRA every 30 min. Standardized meals were consumed during each condition. Study conditions were monitored by accelerometer. | The outcomes were blood pressure (BP) and plasma noradrenaline. Resting BP was measured hourly (3 recordings 20 min post activity) and mean plasma noradrenaline was measured at 30-min intervals for the first hour after meals and hourly thereafter. | The LW and SRA conditions produced significantly (P<.001) reduced resting SBP, DBP, mean plasma noradrenaline, and mean resting heart rate (LW only) compared with sitting control. |  |
|  | **(Dempsey et al., 2017)**  **[27]** | To investigate the effect of interrupting prolonged sitting with brief bouts of light-intensity walking (LW) or simple resistance activities (SRA) on 22h glucose homeostasis in adults with T2D | N=24, mean age 62 ±6, 10 women [41.67%], mean BMI 33.0 | Randomized crossover design | Three 8h conditions on separate days (with washout period): uninterrupted sitting (control), sitting plus 3-min bouts of LW (3.2 km/h) every 30 min, and sitting plus 3-min bouts of SRA every 30 min. Standardized meals were consumed during each condition. Study conditions were monitored by accelerometer. | The outcome was 22h glucose control measured by continuous glucose monitoring recording interstitial fluid glucose concentrations every 5 min for 22h. | The LW and SRA conditions produced significantly (P<.001) reduced 22h glucose and nocturnal mean glucose concentrations and waking glucose compared with sitting control. |  |
|  | **(Grace et al., 2017)**  **[28]** | To investigate the effects of regular interruptions to sitting, compared with prolonged sitting, on the postprandial plasma lipidome | N=21, mean age 63 ±6, 8 women [38.01%], mean BMI 32.7 | Randomized crossover design | Three 8h conditions on separate days (with washout period): uninterrupted sitting (control), sitting plus 3-min bouts of LW (3.2 km/h) every 30 min, and sitting plus 3-min bouts of SRA every 30 min. Standardized meals were consumed during each condition. Study conditions were monitored by accelerometer. | Outcomes include 338 lipid species analysed using mass spectrometry. Outcomes were measured at baseline and after the 8h period. | The LW and SRA conditions produced significantly (P<.05) attenuated elevations in diacylglycerols, triacylglycerols, and phosphatidylethanolamines compared with sitting control.   The light walking and simple resistance activity conditions produced significantly (P<.05) attenuated reductions in plasmalogens, lysol alkyl phosphatidylcholines and phosphatidylserines (LW only) compared with sitting control. |  |
| **3** | **(Duvivier et al., 2017)**  **[29]** | To investigate the effects of breaking sitting with standing and light-intensity walking (LW) vs an energy-matched bout of structured exercise on 24 h glucose levels and insulin resistance in patients with T2D. | N=19, mean age 63 ±9, 6 women [31.58%], mean BMI 30.5 | Randomised crossover design | Three 4day activity regimens (with washout period): sitting (14h of waking day sitting), exercise (1h of sitting time replaced with MVPA) and sit less (4h sitting replaced with 2h LW and 3h standing, sitting broken up every 30 min). Standardized meals were consumed during each condition. Study conditions were monitored by accelerometer. | An outcome was 24h glucose (iAUCs) measured by continuous 24h glucose monitoring on day 4 and blood glucose measurements 4 times per day on day 4. Another outcome was insulin resistance and lipids control measured by blood measurement for glucose, insulin, and lipid measurements after each regimen on day 5. | The exercise condition produced significantly (P<0.05) improvements in glucose, triacylglycerol, NEFA, and energy expenditure over the sitting condition.   The sit-less condition produced significantly (P<0.05) improvements in insulin, c-peptide, triacylglycerol, non-HDL-C, NEFA, 24h glucose AUC, and energy expenditure over the sitting condition.   The sit-less condition produced significantly (P<0.05) improvements in insulin and energy expenditure over the exercise condition. | High |
| **4** | **(Homer, Taylor, Dempsey, Wheeler, Sethi, Townsend, et al., 2021)**  **[30]** | To investigate the effect of interrupting prolonged sitting with simple resistance activities (SRA) at different frequencies on net incremental areas under the curve (iAUC net) for postprandial glucose, insulin, and triglycerides in adults with T2D | N=23, mean age 62 ±8, 10 women [41.67%], mean BMI 32.7 | Randomized crossover design | Three 8h conditions on separate days (with washout period): uninterrupted sitting (control), sitting plus 3-min bouts of SRA every 30 min, sitting plus 6-min bouts of SRA every 60 min. Standardized meals were consumed during each condition. Activity intensity during the interruptions was monitored with activPAL4. | The outcomes were net incremental areas under the curve (iAUC net) for postprandial glucose, insulin, and triglycerides. Blood was collected half-hourly. | The SRA6 conditions produced significantly (P<.05) attenuated glucose and insulin compared with sitting control and SRA3 (glucose only). No significant differences in glucose or insulin were observed in comparison of SRA3 and sitting control. No significant differences of condition on triglyceride were observed. | Some Concerns |
|  | **(Homer, Taylor, Dempsey, Wheeler, Sethi, Grace, et al., 2021)**  **[31]** | To investigate the effect of interrupting prolonged sitting with simple resistance activities (SRA) at different frequencies on 22h glycemic control in adults with T2D | N=24, mean age 62 ±8, 11 women [41.67%], mean BMI 32.7 | Randomized crossover design | Three 8h conditions on separate days (with washout period): uninterrupted sitting (control), sitting plus 3-min bouts of SRA every 30 min, sitting plus 6-min bouts of SRA every 60 min. Standardized meals were consumed during each condition. Activity intensity during the interruptions was monitored with activPAL4. | The outcomes were 22h glycemic control as measured by AUC total, mean glucose, and time in hyperglycemia. Flash glucose monitors assessed glycemic control over a 22h period. | No significant differences of condition on overall 22h glycemic control (AUC total), mean glucose, and time in hyperglycemia were observed. |  |
|  | **(Taylor et al., 2021)**  **[32]** | To investigate the acute effects of interrupting prolonged sitting with simple resistance activities (SRA) at different frequencies on vascular function in T2D. | N=24, mean age 61.5 ±7.8, 11 women [45.83], mean BMI 32.6 | Randomized crossover design | Three 7h sitting conditions on separate days (with washout period): uninterrupted sitting (SIT), sitting with 3-min bouts of SRA every 30min (SRA3), and sitting with 6-min bouts of SRA every 60min (SRA6). Study conditions were monitored by activPAL. Standardized meals were consumed during each condition. | Outcomes include femoral artery flow-mediated dilation (FMD), resting shear rate, blood flow, and endothelin-1 measured at 0, 1, 3.5, 4.5, and 6.5-7h. | SRA3 produced significantly higher FMD (P=.04) over 7h compared to SIT.   SRA3 and SRA6 produced significantly (P<.001 and P<.001 respectively) higher mean resting femoral shear rate over 7h compared to SIT.  Endothelin-1 concentrations were not statistically different between conditions. |  |
| **5** | **(Honda et al., 2016)**  **[33]** | To investigate the effect of interrupting SB by a 3-min bout of stair climbing at 60min and 120min on postprandial blood glucose in adults with T2D. | N=16, mean age 65 ±1.1, 3 women [18.75%], mean BMI 23.6 | Randomized crossover design | Two 180min postprandial condition on separate days (with washout period): uninterrupted postprandial sitting (control) and interrupted postprandial sitting with 3 min bout of stair climbing descending exercise (ST-EX) at 60min and 120min. Standardized meals were consumed during each condition. Heart rate was recorded using a Polar Accurex Plus monitor and perceived exertion was recorded using the Bord's rating scores. | The outcomes were postprandial blood glucose (BG) response. Capillary BG was collected by capillary blood samples before (0min), immediately before the first ST-EX (60min), 90min, immediately before the second ST-EX (120min), 150min, and 18-min after the meal. | No significant differences of condition on BG were found at 60min.   The ST-EX condition had significantly (P<.01) lower BG at 150min than control.  The ST-Ex condition had significantly lower area under the curve (P<.05) compared to control. | Some Concerns |
| **6** | **(Paing et al., 2019a)**  **[34]** | To investigate the dose–response between frequency of interruption of sedentary time and basal glucose in T2D | N=12 (n=8/condition), mean age 60.0 ±3.2, 4 women [33.33%], mean BMI 30.2 | Randomized three-treatment, two-period cross over design | Three 7h sitting conditions on separate days (with washout period): interrupted every 60 min (condition 1), interrupted every 30 min (condition 2), and interrupted every 15 min (condition 3) by 3-min bouts of LW. Study conditions were monitored by accelerometer (activPAL3). Standardized meals were consumed during each condition. | Outcomes include changes in fasting glucose, magnitude and duration of the dawn phenomenon, night-time mean glucose and time glycaemic variability of the nights and early mornings before and after treatment conditions measured by continuous 24h glucose monitoring. | Condition 3 produced significantly (P<.05) improved fasting glucose and duration of the dawn phenomenon compared with condition 1.  Condition 3 produced significantly (P<.05) improved magnitude of the dawn phenomenon compared with condition 2.  Condition 3 produced significantly (P<.05) improved night-time glycaemic variability compared with condition 1 and 2. | Some Concerns |
|  | **(Paing et al., 2019b)**  **[35]** | To investigate the dose–response between frequency of interruption of sedentary time and glucose control in T2D | N=12 (n=8/condition), mean age 60.0 ±11, 4 women [33.33%], mean BMI 30.2 | Randomized three-treatment, two-period cross over design | Three 7h sitting conditions on separate days (with washout period): interrupted every 60 min (condition 1), interrupted every 30 min (condition 2), and interrupted every 15 min (condition 3) by 3-min bouts of LW. Study conditions were monitored by accelerometer (activPAL3). Standardized meals were consumed during each condition. | Outcomes include postprandial glucose incremental area under the curves (iAUCs) and 21-h glucose total area under the curve (AUC) measured by continuous 24h glucose monitoring. | Condition 3 produced significantly (P<.04) improved post-breakfast glucose iAUC compared with Condition 1.   Condition 3 and condition 2 produced significantly (P<.03 and P<.05 respectively) improved post-lunch iAUC compared to Condition 1.   Condition 3 and Condition 2 produced significantly (P=.02 and P<.04 respectively) improved post-dinner glucose iAUC compared with Condition 1.   Condition 3 produced significantly (P=.02) improved postprandial glucose iAUC compared with Condition 1.  Condition 3 produced significantly (P<.001 and P=.002 respectively) improved 21-h glucose AUC compared with Condition 1 and Condition 2.  Condition 3 was not produced significant (P≥.05) improved post-breakfast glucose iAUC, cumulative 10.5-h postprandial glucose iAUC, and 21-h glucose AUC compared to Condition 2 and Condition 1. |  |
| **7** | **(Van Dijk et al., 2013)**  **[36]** | To investigate the impact of activities of daily living (ADL) versus MVPA on 24-h glycemic control in patients with T2D | N=20, mean age 64 ±1, women n=0 [0%], mean BMI 29.5 | Randomized crossover design | Three 3d conditions on separate days (with washout period): SB control, breaking up SB by three post-meal 15-min bouts of ADL (3 METs), and breaking up SB by a single 45-min bout of MVPA (6 METs) performed on day 2. Standardized meals were consumed during each condition. | Outcomes included blood glucose concentrations assessed by 24h continuous glucose monitoring, and plasma insulin concentrations assessed by frequently sampled venous blood. | The ADL and exercise conditions produced significantly (P<.05) improvements in glucose and insulin compared with the sedentary condition. However, the amount of time hyperglycemic was only significantly (P<.05) reduced in the exercise condition compared with the SB condition | Some Concerns |
