# Supplementary material for: Interventions to reduce sedentary behaviour in adults with type 2 diabetes: A systematic review and meta-analysis
Source: PLoS One. 2024 Jul 30;19(7):e0306439. doi: 10.1371/journal.pone.0306439 (PMC11288443; doi:10.1371/journal.pone.0306439)
Supplement: S2 Table — (DOCX) [file pone.0306439.s002.docx]

Table 2. Longer-term SB interventions in adults with T2D

|  | **Study** | **Objective** | **Participants** | **Research Design** | **Intervention/ Comparison** | **SB Outcomes** | **Cardio Metabolic Outcomes** | **Main Results** | **RoB2**  **Quality** |
| --- | --- | --- | --- | --- | --- | --- | --- | --- | --- |
| **1** | **(Alonso-Domínguez et al., 2019)**  **[37]** | To investigate the short and long-term impact of a multifactorial intervention on PA and clinically relevant biochemical parameters in patients with T2D mellitus. | N=204 (n=102/ condition), control group: mean age 60.4 ±8.4, 41 women [40.2%], mean BMI 30.3, intervention group: mean age 60.8 ±7.8, 52 women [51.1%], mean BMI 29.5 | Randomized control trial | Two 3-month groups (intervention and control) with a 12-month follow-up. Both groups received standardized counselling for 10 minutes on PA and diet. The intervention group received an additional multifactorial intervention consisting of 5 heart-healthy walks, use of a smartphone app, and a diet workshop. | The secondary outcome sedentary time (min/day) was assessed by the IPAC at baseline, 3 months, and 12 months. | The secondary cardiometabolic outcomes blood pressure, body mass index, and biochemical parameters (fasting plasma glucose, glycated haemoglobin, triglycerides, total serum cholesterol, low-density lipoprotein cholesterol and high-density lipoprotein-cholesterol levels) were collected at baseline, 3 months, and 12 months. | Sedentary time significantly decreased for the intervention group at 3-months (p<.001) and 12-months (p<.001).   Sedentary time was significantly different between the control and intervention group at 3-months (p<.05) but not at 12-months (p>.05).   Postprandial glycaemia, lipid profile, and systolic blood pressure significantly (p<.05) improved for the intervention group at 3-months. LDL cholesterol, anthropometric parameters, and systolic blood pressure significantly (p<.05) improved for the intervention group at 12-months.   BMI and waist circumference were significantly different (p<.05) between the control and intervention groups at 3-months. No significant differences were found between the groups at 12 months (p>.05). | Some Concerns |
| **2** | **(Alothman et al., 2021)**  **[38]** | To investigate the feasibility and effectiveness of SB counseling and activity monitoring on decreasing SB, increasing PA, and glycemic control in older adults with T2D. | N = 10, mean age 65.6±7.3, 8 women [80.0%], mean BMI 32.7 | Pilot pre-post intervention design | One (intervention group) 13-week intervention. The intervention group received in person SB counseling (self-efficacy and motivational interviewing) at the end of weeks 1, 5 and 9. The intervention group wore an activity monitor at weeks 1, 5, 9, and 13 with vibrotactile features (after 20 min sitting) enabled for 7 days at weeks 5 and 9. | The outcome siting time (hours/day), step count (step/day), and standing time (hours/day) was assessed by activPAL3TM at weeks 1, 5, 9, and 13. | The outcome glycemic control was assessed at weeks 1 and 13 by HbA1c. | Sitting time and step count significantly decreased from baseline to post-intervention (p<0.05).  Standing time did not significantly decreased from baseline to post-intervention (p>0.05).  HbA1c significantly decreased from baseline to post-intervention (p<0.05). | High |
| **3** | **(Balducci et al., 2017)**  **[39]** | To investigate the efficacy of a behavioural intervention strategy in increasing total daily PA and reducing sedentary time in patients with T2D compared to standard care. | N=300 (150/ group), control group: mean age 62.3 ±10.1, 57 women [38.0%], mean BMI 30.1, intervention group: 61.0 ±9.7, 59 women [39.3%], mean BMI 30.0 | Randomized control trial | Two 3-year groups (intervention and control). The intervention group received the behavioral intervention consisting of 1 individual theoretical counseling session and 8 individual biweekly theoretical and practical counseling sessions each year for 3 years. The control group received standard care for 3 years. | The outcome sedentary time was assessed by accelerometer at baseline for 4 months and every 4 months for 7-day until the end of year 3. | Secondary cardiometabolic outcomes included BMI, waist circumference, body composition, blood pressure, HBA1C, fasting plasma glucose, serum insulin, triglycerides, cholesterol, hs-CRP, serum creatinine, and albumin-to-creatinine ratio. All parameters were obtained at baseline and every 4 months until the end of year 3. | Sedentary time significantly decreased for the intervention group from baseline to 4 months (p<.0001), for the control group from baseline to 4 months (p<.0001), and significantly more for the intervention group compared to control (p<.0001).  HbA1c significantly decreased for the intervention group from baseline to 4 months (p<.0001). Fat mass significantly increased for the control group form baseline to 4 months (p<.0001). There were no other significant cardiometabolic changes for the intervention group from baseline to 4 months, for the control group from baseline to 4 months, and between the intervention and control groups. | Some Concerns |
|  | **(Balducci et al., 2019)**  **[40]** | To investigate whether a behavioral intervention strategy can produce a sustained increase in PA and reduction in SB among individuals with T2D | N=300 (150/ group), control group: mean age 62.3 ±10.1, 57 women [38.0%], mean BMI 30.0, intervention group: 61.0 ±9.7, 59 women [39.3%], mean BMI 30.1 | Randomized control trial | Two 3-year groups (intervention and control). The intervention group received the behavioral intervention consisting of 1 individual theoretical counseling session and 8 individual biweekly theoretical and practical counseling sessions each year for 3 years. The control group received standard care for 3 years. | The outcome sedentary time was assessed by accelerometer at baseline for 4 months and every 4 months for 7-day until the end of year 3. | Secondary cardiometabolic outcomes included BMI, waist circumference, body composition, blood pressure, HBA1C, fasting plasma glucose, serum insulin, triglycerides, cholesterol, hs-CRP, serum creatinine, and albumin-to-creatinine ratio. All parameters were obtained at baseline and every 4 months until the end of year 3. | Participants in the behavioral intervention and standard care groups accumulated, respectively, 10.9 vs 11.7 h/d of sedentary time (difference, −0.8 [95% CI, −1.0 to −0.5]; P < .001).  The mean difference in participants in the behavioral intervention vs standard care group was significant for fasting plasma glucose (P = .007), systolic blood pressure (P = .02), total CHD 10-year risk score (P = .03), and fatal CHD 10-year risk score (P = .04) over time, whereas the differences did not achieve statistical significance for the remaining cardiovascular risk factors and scores.   The between-group mean difference in change from baseline was significant for total stroke risk score (P = .04) after 2 years and HbA1c (P = .02), fasting plasma glucose (P = .04), 10-year total CHD risk score ( P= .01), 10-year fatal CHD risk score (P = .008),0-year total CHD ( P= .01), fatal CHD (P = .008), and total stroke (P = .01) after 3 years. |  |
|  | **(Balducci et al., 2022a)**  **[41]** | A post-hoc analysis to investigate the behavioural intervention (and associated changes in PA/SB) on estimated B-cell function and insulin sensitivity in individuals with T2D. | N=203 (excluded participants who did not complete the study N=33 and those on insulin N=64), control group N=98: mean age 62.8±9.9, 37 women [37.8%], mean BMI 29.5, intervention group N=105: 60.8 ±9.8, 40 women [38.1%], mean BMI 29.9 | Randomized control trial | Two 3-year groups (intervention and control). The intervention group received the behavioral intervention consisting of 1 individual theoretical counseling session and 8 individual biweekly theoretical and practical counseling sessions each year for 3 years. The control group received standard care for 3 years. | The outcome sedentary time was assessed by accelerometer at baseline for 4 months and every 4 months for 7-days until the end of year 3.  Participants in the INT group were stratified by baseline to end-of-study changes in SB, MVPA, LPA, and PA volume in 3 groups, values worsened or remained substantially stable (Group 1), improved moderately (Group 2), or improved markedly (Group 3). | Baseline and end-of-study β-cell function and insulin sensitivity were estimated by the revised version of the HOMA method. | The mean difference in participants in the behavioral intervention vs standard care group was significant (p<0.05) for HOMA-B%_ins_, HOMA-B%_c-pep_, HOMA-S%_c-pep_, HOMA-IR_c-pep_ QUICKI, DI_ins_, and DI_c-pep_ over time, whereas the differences did not achieve statistical significance for HOMA-S%_ins_ and HOMA-IR_ins_,  Among INT participants, baseline to end-of-study changes in HOMA-B%_ins_, HOMA-B%_c-pep_ and DI_ins_, and DI_c-pep_ increased from Group 1 to Group 3 of SB.  Upon univariate analysis, baseline to end-of-study changes in HOMA-B%_ins_, HOMA-B%_c-pep_ and DI_ins_, and DI_c-pep_ correlated significantly with decreases in SB.  When removing change in MVPA from the models, change in SB was independently associated with DI_ins_ and DI_c-pep._ |  |
|  | **(Balducci et al., 2022b)**  **[42]** | A post-hoc analysis to investigate the relationship of changes in PA and SB with changes in physical fitness and CVD risk in individuals with T2D. | N=300 (150/ group), control group: mean age 62.3 ±10.1, 57 women [38.0%], mean BMI 30.0, intervention group: 61.0 ±9.7, 59 women [39.3%], mean BMI 30.1 | Randomized control trial | Two 3-year groups (intervention and control). The intervention group received the behavioral intervention consisting of 1 individual theoretical counseling session and 8 individual biweekly theoretical and practical counseling sessions each year for 3 years. The control group received standard care for 3 years. | The outcome sedentary time was assessed by accelerometer at baseline for 4 months and every 4 months for 7-days until the end of year 3.  Changes  from baseline over the 3-year  period in total PA volume, LPA,  MVPA, and SB  stratified  by quartiles. | Changes from baseline over the 3-year  period in physical fitness  and modifiable CVD factors and  scores stratified by quartiles. | SB quartile I (+0.72 h/day), II (~no change), III (-0.60 h/day), and IV (-1.53 h/day).  Results for SB and CVD factors are reported:  Upon univariate analysis, changes in SB correlated significantly with HbA1c, FPG, hs-CRP, total and fatal CHD 10-risk, BMI and total stroke 10-risk score. In model 1, changes in SB was an independent predictors of change in HbA1c. When MVPA change was removed from the model, change in  SB became an independent predictor of triglycerides. Only change in SB was an independent predictor of change FPG, diastolic BP, and CHD 10-year risk scores and, inversely, of change in HDL cholesterol. |  |
| **4** | **(Bailey, Mugridge, Dong, Zhang, & Chater, 2020)**  **[43]** | To evaluate the feasibility and acceptability of a self-regulation smartphone app for reducing prolonged sitting in people with T2D mellitus. | N=18 (9/ group), control group: mean age 55 ±6, 3 women [6.66%], mean BMI 29.9, intervention group: mean age 57 ± 7, 6 women [3.33%], mean BMI 31.1 | Randomized control trial | Two 8-week groups (intervention and control). The intervention group used the MyHealthAvatar-Diabetes smartphone app for 8 weeks that used several behavioural change techniques aimed at reducing and breaking up sitting time. | The outcomes sitting time and breaks in sitting per day were monitored in real-time, using a combination of the smartphone's built-in-accelerometers and gyroscope sensor. Participants were also asked to wear an activPAL at week 0 and 8. | Secondary cardiometabolic outcomes included weight, body fat %, body mass index, waist circumference, heart rate, systolic blood pressure, diastolic blood pressure, fasting blood glucose, 2-h blood glucose. | From baseline to follow-up participants increased the number of breaks per day (large effect in favour of the intervention group). Small effects were found for sitting time.  There was a medium effect for the change in body fat % and 2-h blood glucose in favour of the intervention group. The effect sizes for the between group differences were trivial or small for the remaining cardiometabolic outcomes. | High |
| **5** | **(Brazo-Sayavera et al., 2021)**  **[44]** | To evaluate the effectiveness of a multicomponent supervised and unsupervised training program on PA, SB, and health related quality of life in adults with T2D. | N = 35, control group: N = 14, mean age 73.1 ± 3.9, women [57.1%], mean BMI 28.3, intervention group: N = 21, mean age 74.7 ± 4.5, women [66.7%], mean BMI 30.03 | Nonrandomized control trial | Two 11-week groups (intervention and control). The intervention group received 5 weeks of supervised power training followed by 6 weeks of unsupervised home-based power training. The control group continued their usual lifestyle and medical care. | The outcome sedentary time was assessed by self-reporting their average number of sitting hours at baseline and post the 11-week intervention. |  | The sitting time increased by 58% and 32% for control group (d = −1.84) and intervention group (d = −1.17), respectively (time × group interaction, P < .05). | High |
| **6** | **(Connelly et al., 2017)**  **[45]** | To develop and explore the feasibility and effect of a Web-based PA promotion intervention in people with T2D. | N = 31, written information control group: N = 10, mean age 66.5 ± 6.0, women 6 [60.0%], mean BMI 31.4, web information control group: N = 10, mean age 66.2 ± 8.4, women 2 [20.0%], mean BMI 33.0, interactive web intervention group: N = 11, mean age 67.3 ± 10.4, women 5 [45.5%], mean BMI 30.3 | Mixed method pilot randomised control trial | Three 6-month groups (1 - written information control group, 2 - web information control group, and 3 - interactive web intervention group).  Group 1 received leaflets based on the website material. Group 2 were given online access to diabetes specific PA information, but not interactive features. Group 3 had online access to diabetes-specific PA with information and interactive features. | The secondary outcome sedentary time (min/wk) was assessed by 7-day accelerometer at baseline, 3 months, and 6 months. | The secondary outcome HbA1c was assessed at baseline, 3 months, and 6 months. | Group 1: ST decreased from 2993 (SD 662) to 2429 (SD 776) min/week at 3 months, followed by an increase to 2833 (SD 764) min/week at 6 months.  Group 2: ST decreased from 3202 (SD 759) to 3129 (SD 997) min/week at 3 months, further decreasing to 3055 (SD 807) min/week at 6  months.  Group 3: ST decreased from 3275 (SD 646) to 2509 (SD 756) min/week at 3 months, increasing to 3004 (SD  485) min/week at 6 months.  Group 1 HbA1c dropped from 55.3 (SD 13.7) to 54.4 (SD 15.6) mmol/mol at 3 months, further decreasing to 50.5 (SD 5.9) mmol/mol at 6 months.  Group 2 increased from  52.4 (SD 8.2) to 55.8 (SD 8.0) mmol/mol at 3 months, then decreased to 55.0 (SD 4.7) mmol/mol at 6 months.  Group 3 HbA1c decreased from 57.7 (SD 11.2) to 56.7 (SD 10.0) mmol/mol at 3 months, further decreasing to 54.1 (SD 9.5) mmol/mol at 6 months. | Some Concerns |
| **7** | **(De Greef, Deforche, Tudor-Locke, & De Bourdeaudhuij, 2010)**  **[46]** | To investigate the benefits of a pedometer and a cognitive-behavioural group intervention for promoting PA in T2D patients | N=41, mean age 61.3 [6.5] years, 13 women [31.71%], mean BMI 30.2 | Randomized control trial | Two 12-week groups (intervention and control) with a 1-year follow-up. The intervention group (n=20) received five cognitive-behavioural group sessions of 90 min within 12 weeks, a booster session after 22 weeks and a pedometer. The control group (n=21) received usual care, a single educational session, and no pedometer. | The primary outcome was PA (including SB) assessed by accelerometer and pedometer. Outcomes were measured at baseline (T0, week 1), post-intervention (T1, week 13), and at follow up (T2, week 52). | Secondary outcomes were weight, body mass index, blood pressure, haemoglobin A1c and total cholesterol. Outcomes were measured at baseline (T0, week 1), post-intervention (T1, week 13), and at follow up (T2, week 52). | Participants in the behavioral intervention had significant (P<.05) increased step count and reduced SB from baseline to post-intervention. However, no significant differences were found at follow up. There were no other significant intervention effects. However, participants had significant (P<0.05) improved step count, PA, SB, HbA1c, and diastolic BP from baseline to follow up. | Some Concerns |
| **8** | **(De Greef et al., 2011)**  **[47]** | To investigate the effectiveness of a behavioral modification program on PA and SB in diabetes patients | N=92, mean age 62 [9] years, 29 women [31%], mean BMI 30.0 | Randomized control trial | Two 24-week groups (intervention and control) with a 1-year follow-up. The intervention group (n=60) received 24 week cognitive-behavioral therapy intervention consisting of a 30 min face-to-face session, pedometer and seven 20 min telephone follow-ups. The control group(n=32) received usual care. | The outcomes were PA and SB measured by pedometer (steps/day), accelerometer (min/day) and interview based International PA Questionnaire (IPAQ) (min/day). Outcomes were measured at baseline, immediately after the 24-week intervention and 1 year after baseline. |  | Participants in the behavioral intervention had significantly (P<.05) increased their steps/day, their total PA, and decreased their SB after the 24-week intervention and 1 year after baseline. | High |
| **9** | **(Hsu et al., 2023)**  **[48]** | To investigate the effects of short bouts of walking on HbA1c and HOMA-IR in older adults with type 2 diabetes | N=48, control: N=12, mean age 63±6.7, women 6 [50%], mean BMI 25.3, 10 000 steps daily intervention: N=13, mean age 61.3±6.8, women 6 [46.2%], mean BMI 26.4, 10-min bout of walking intervention: N=13, mean age 60.5±8.6, women 7 [53.8%], mean BMI 27.9 | Randomized control trial | Three 12-week groups (control, 10 000 step intervention [10k], and 10-min bout of walking intervention [10/100MW]).  All participants wore a pedometer, were asked to maintain their normal diet, and continue their medications. According to dietary logs, total kcal among the 3 groups were similar (p>0.05).  The 10k intervention group received text-message reminders to walk in their spare time.  The 10/100MW group had three walking bouts: morning, afternoon, and evening, and received text-message reminders to walk in their spare time.  The control group were asked to maintain their normal lifestyle. |  | ­HbA1c, HOMA-IR, and lipids (TG, LDL-C, HDL-C, and TC) were measures pre and post intervention. | Th e rate of completing 10,000 steps daily for  the 10KS group was 100%. In the 10/100MW group, the  rate of completing three 10-minute bouts of walking at  100 steps/min was >95%.  Average daily steps in the 10KS group during the final week (10,613 [SD = 811] steps/day) were significantly greater than those in the 10/100MW and CON groups (7,623 [SD = 536] steps/day and 4,365 [SD = 429] steps/day, respectively; p < 0.05)  HbA1c improve post intervention in the 10K and 10/100MW groups (p<0.05) and in the 10K and 10/100MW groups compared to control (p<0.05).  HOMA-IR improve post intervention in the 10K and 10/100MW groups (p<0.05) and in the 10K and 10/100MW groups compared to control (p<0.05).  TG improve post intervention in the 10K and 10/100MW groups (p<0.05) and in the 10K group compared to control (p<0.05).  HOMA-IR improve post intervention in the 10K and 10/100MW groups (p<0.05) and in the 10K and 10/100MW groups compared to control (p<0.05)  LDL-C and HDL-C were not significantly different overtime or between groups (p>0.05). | Some Concerns |
| **10** | **(Jennings et al., 2013)**  **[49]** | To investigate the effectiveness of a 12-week theory and web-based program to increase PA in adults with Type 2 diabetes. | N=397, control: N = 202, mean age 58.3 ± 9.9, women 95 [47.0%], mean BMI 33.6, intervention: N = 195, mean age 58.2 ± 10.6, women 94 [48.2%], mean BMI 33.5 | Randomized control trial | Two 12-week groups (intervention and control) with a 6-month follow-up. The intervention group (n=195) received a 12-week theory and web-based programme with weekly modules grounded in the theory of planned behaviour and a pedometer. The control group (n=202) received modified version of the wed based programme and a pedometer. | The secondary outcome (weekday and weekend) sitting time (min/day) was assessed by the IPAC at baseline, 12 weeks, and 36 weeks. |  | ITT analysis demonstrated significant time effects for all  ST measures (p<0.05). There were no group by time interactions.  The completer's analysis showed that there was a significant time effect for all ST measures (p<0.05). There were significant group by time interactions for weekday sitting time (p<0.05), where the intervention group significantly decreased ST in comparison to the control group. | High |
| **11** | **(Miyamoto, Fukunda, Oshima, & Moritani, 2017)**  **[50]** | To investigate whether the use of a tri-axial accelerometer can reduce SB and increase non-locomotive PA (N-LPA), and to investigate the effect of this intervention on parameters of glucose and fat metabolism in patients with T2D | N=31, mean age 60.7 ±1.6, mean BMI 24.7 | Randomized control trial | Three 12-week groups: N-LPA (non-locomotive PA), LPA (locomotive PA), and control. Patients in the N-LPA group received verbal instructions to increase their N-LPA and patients in the LPA group received verbal instruction to increase their LPA at week 0, 4, and 8. Patients in the N-LPA and LPA groups wore tri-axial accelerometer with the display turned on for the 12-weeks, so they received visual feedback about their activity levels. Participants in the control group were given no instructions and were asked to wear the tri-axial accelerometer with the display turned off for the 12-weeks, so they received no visual feedback about their activity levels. All participants provided 24-h dietary written recall for any 2 days within the last 10-day period before the start of the experiment (baseline) and 12-week (follow-up) examination. The 24-h dietary written recall was analyzed by a nutritionist to calculate average energy intake per day. | The outcomes included SB accessed by tri-axial accelerometer. | The outcomes included glucose metabolism (fasting glucose and HbA1c) and fat metabolism (body mass, triglyceride, low-density lipoprotein, cholesterol, and total cholesterol) measured pre and post intervention. | The N-LPA intervention significantly (p < 0.05) reduced SB and increased N-LPA over time. These changes did not significantly improve glucose and fat metabolism. However, a significant (p < 0.05) positive correlation between the change in SB and HbA1c levels was found. | High |
| **12** | **(Poppe et al., 2019)**  **[51]** | To either increase PA or to decrease SB (participants decided) in adults with T2D (RCT 1) or ≥50 years (RCT 2). | RCT 1: N=54, mean age 62.67 ±8.40, 20 women [37.03%], mean BMI 30.84 - 12 allocated to SB and 24 allocated to PA | Randomized controlled trial | Two 5-week groups (intervention and waitlist control). The intervention group received 5 mobile app sessions. HAPA sessions with an interval of 1 week between each session. | LASA questionnaire measured the total sitting time (min/day) pre and post intervention. The Accelerometer measured the number of breaks per day, length of sedentary bouts (min/day), and sedentary time (min/day). |  | A significant intervention effect favoring the SB intervention group was found for accelerometer assessed daily breaks from sedentary time (P=.005).   No significant intervention effects were found for the SB intervention group for the LASA questionnaire measured the total sitting time (min/day) or accelerometer measured length of sedentary bouts (min/day) and sedentary time (min/day). | High |
