# Supplementary material for: Interventions to reduce sedentary behaviour in adults with type 2 diabetes: A systematic review and meta-analysis
Source: PLoS One. 2024 Jul 30;19(7):e0306439. doi: 10.1371/journal.pone.0306439 (PMC11288443; doi:10.1371/journal.pone.0306439)
Supplement: S3 Table — (DOCX) [file pone.0306439.s003.docx]

### **S3 Table. Quality of eligible interventions.**

| **Study** | | **Domain 1** | | **Domain 2** | | **Domain 3** | **Domain 4** | **Domain 5** | **Overall Risk of Bias** |
| --- | --- | --- | --- | --- | --- | --- | --- | --- | --- |
|  |  | Risk of bias arising from the randomization process | Risk of bias arising form period and carryover effects | Risk of bias due to deviations from the intended intervention (effect of assignment to intervention) | Risk of bias due to deviations from the intended interventions (effect of adhering to intervention) | Missing outcome data | Risk of bias in measurement of the outcome | Risk of bias in selection of the reported result |  |
| **Short-Term Crossover** | | | | | | | | | |
| **1** | **(Blankenship et al., 2019)**  **[24]** | Some Concerns | Low | Some Concerns | Low | Low | Low | Some Concerns | Some Concerns |
| **2** | **(Dempsey, Larsen, et al., 2016)**  **[25]** | Low | Low | Some Concerns | Low | Low | Low | Low | Some Concerns |
|  | **(Dempsey, Sacre, et al., 2016)**  **[26]** |  |  |  |  |  |  |  |  |
|  | **(Dempsey et al., 2017)**  **[27]** |  |  |  |  |  |  |  |  |
|  | **(Grace et al., 2017)**  **[28]** |  |  |  |  |  |  |  |  |
| **3** | **(Duvivier et al., 2017)**  **[29]** | Low | Low | Some Concerns | High | Low | Low | Low | High |
| **4** | **(Homer, Taylor, Dempsey, Wheeler, Sethi, Townsend, et al., 2021)**  **[30]** | Low | Low | Some Concerns | Some Concerns | Low | Low | Low | Some Concerns |
|  | **(Homer, Taylor, Dempsey, Wheeler, Sethi, Grace, et al., 2021)**  **[31]** |  |  |  |  |  |  |  |  |
|  | **(Taylor et al., 2021)**  **[32]** |  |  |  |  |  |  |  |  |
| **5** | **(Honda et al., 2016)**  **[33]** | Some Concerns | Low | Some Concerns | Low | Low | Low | Some Concerns | Some Concerns |
| **6** | **(Paing et al., 2019a)**  **[34]** | Low | Low | Some Concerns | Some Concerns | Low | Low | Low | Some Concerns |
|  | **(Paing et al., 2019b)**  **[35]** |  |  |  |  |  |  |  |  |
| **7** | **(Van Dijk et al., 2013)**  **[36]** | Some Concerns | Low | Some Concerns | Some Concerns | Low | Low | Some Concerns | Some Concerns |
| **Longer Term RCT** | | | | | | | | | |
| **1** | **(Alonso-Domínguez et al., 2019)**  **[37]** | Low | N/A | Some Concerns | Some Concerns | Some Concerns | Some Concerns | Low | Some Concerns |
| **2** | **(Althoman et al., 2021)**  **[38]** | High | N/A | Some Concerns | Low | Low | Low | Low | High |
| **3** | **(Balducci et al., 2017)**  **[39]** | Low | N/A | Low | Some Concerns | Low | Some Concerns | Low | Some Concerns |
|  | **(Balducci et al., 2019)**  **[40]** |  |  |  |  |  |  |  |  |
|  | **(Balducci et al., 2022a) [41]** |  |  |  |  |  |  |  |  |
|  | **(Balducci et al., 2022b)**  **[42]** |  |  |  |  |  |  |  |  |
| **4** | **(Bailey et al., 2020)**  **[43]** | Some Concerns | N/A | Some Concerns | High | Low | Some Concerns | Low | High |
| **5** | **(Brazo-Sayavera et al., 2021)**  **[44]** | High | N/A | High | High | High | High | Some Concerns | High |
| **6** | **(Connelly et al., 2017)**  **[45]** | Some Concerns | N/A | Some Concerns | Some Concerns | Some Concerns | Low | Low | Some Concerns |
| **7** | **(De Greef et al., 2010)**  **[46]** | Low | N/A | Some Concerns | Some Concerns | Low | Low | Some Concerns | Some Concerns |
| **8** | **(De Greef et al., 2011)**  **[47]** | Low | N/A | Some Concerns | High | Low | Low | Some Concerns | High |
| **9** | **(Hsu et al., 2023)**  **[48]** | Some Concerns | N/A | Some Concerns | Some Concerns | Low | Low | Some Concerns | Some Concerns |
| **10** | **(Jennings et al., 2014)**  **[49]** | ­­Low | N/A | Low | High | High | High | High | High |
| **11** | **(Miyamoto et al., 2017)**  **[50]** | Low­­­ | N/A | Some Concerns | Some Concerns | High | Low | Some Concerns | High |
| **12** | **(Poppe et al., 2019)**  **[51]** | Low | N/A | Some Concerns | High | Some Concerns | Low | Low | High |
