# Supplementary material for: Interventions to reduce sedentary behaviour in adults with type 2 diabetes: A systematic review and meta-analysis
Source: PLoS One. 2024 Jul 30;19(7):e0306439. doi: 10.1371/journal.pone.0306439 (PMC11288443; doi:10.1371/journal.pone.0306439)
Supplement: S1 File — (DOCX) [file pone.0306439.s004.docx]

### **S1 File. Database Searches.**

**PubMed, Sept 16, 2022 = 1899**

**(((((((("sedentary behavior"[MeSH Terms]) OR ("sitting position"[MeSH Terms])) OR ("television"[MeSH Terms])) OR ("sedentary time*"[Title/Abstract])) OR ("sedentary behavior*" [Title/Abstract])) OR ("sedentary behaviour*"[Title/Abstract])) OR ("sedentary lifestyle*"[Title/Abstract])) OR ("screen time*"[Title/Abstract])) AND ((((type 2 diabetes mellitus[MeSH Terms]) OR (diabetes mellitus[Title/Abstract])) OR (type 2 diabet*[Title/Abstract])) OR (non insulin dependent diabet*[Title/Abstract]))**

**PubMed, Jan 3, 2024 = 205**

(((((((("sedentary behavior"[MeSH Terms]) OR ("sitting position"[MeSH Terms])) OR ("television"[MeSH Terms])) OR ("sedentary time*"[Title/Abstract])) OR ("sedentary behavior*" [Title/Abstract])) OR ("sedentary behaviour*"[Title/Abstract])) OR ("sedentary lifestyle*"[Title/Abstract])) OR ("screen time*"[Title/Abstract])) AND ((((type 2 diabetes mellitus[MeSH Terms]) OR (diabetes mellitus[Title/Abstract])) OR (type 2 diabet*[Title/Abstract])) OR (non insulin dependent diabet*[Title/Abstract])) Filters: from 2022/9/16 - 2024/1/3

**Embase, Sept 16, 2022 = 3775**

1 exp sitting/ 27650

2 sedentary lifestyle/ 18285

3 exp sedentary time/ 2132

4 exp screen time/ 2100

5 exp television viewing/ 3640

6 "sedentary behavior*".tw. 6549

7 "sedentary behaviour*".tw. 3870

8 "sedentary lifestyle*".tw. 7190

9 "sedentary time*".tw. 4086

10 1 or 2 or 3 or 4 or 5 or 6 or 7 or 8 or 9 58452

11 exp non insulin dependent diabetes mellitus/ 306688

12 "diabetes mellitus".tw. 332865

13 "non insulin dependent diabet*".tw. 11629

14 "type 2 diabet*".tw. 243951

15 11 or 12 or 13 or 14 570862

16 10 and 15 3775

|  |
| --- |
| \|  \| \| --- \| |

**Embase, Jan 3, 2024 = 739**

1 exp sitting/ 30264

2 sedentary lifestyle/ 21214

3 exp sedentary time/ 2897

4 exp screen time/ 3130

5 exp television viewing/ 4144

6 "sedentary behavior*".tw. 7604

7 "sedentary behaviour*".tw. 4412

8 "sedentary lifestyle*".tw. 8113

9 "sedentary time*".tw. 4688

10 1 or 2 or 3 or 4 or 5 or 6 or 7 or 8 or 9 66334

11 exp non insulin dependent diabetes mellitus/ 345952

12 "diabetes mellitus".tw. 366167

13 "non insulin dependent diabet*".tw. 11738

14 "type 2 diabet*".tw. 274031

15 11 or 12 or 13 or 14 633197

16 10 and 15 4320

17 limit 16 to yr=“2022-Current” 739

**Scopus, Sept 16, 2022 = 1952**

( ( TITLE ( "type 2 diabet*" ) ) OR ( ABS ( "type 2 diabet*" ) ) OR ( ABS ( "non insulin dependent diabet*" ) ) OR ( TITLE ( "non insulin dependent diabet*" ) ) OR ( TITLE ( "diabetes mellitus" ) ) OR ( ABS ( "diabetes mellitus" ) ) ) AND ( ( ABS ( "television viewing" ) ) OR ( TITLE ( "television viewing" ) ) OR ( ABS ( "screen time*" ) ) OR ( TITLE ( "screen time*" ) ) OR ( ABS ( "sitting" ) ) OR ( TITLE ( "sitting" ) ) OR ( ABS ( "sedentary time*" ) ) OR ( TITLE ( "sedentary time*" ) ) OR ( ABS ( "sedentary lifestyle*" ) ) OR ( TITLE ( "sedentary lifestyle*" ) ) OR ( ABS ( "sedentary behavior*" ) ) OR ( TITLE ( "sedentary behavior*" ) ) OR ( ABS ( "sedentary behaviour*" ) ) OR ( TITLE ( "sedentary behaviour*" ) ) )

**Scopus, Jan 3, 2024 = 368**

( ( TITLE ( "type 2 diabet*" ) ) OR ( ABS ( "type 2 diabet*" ) ) OR ( ABS ( "non insulin dependent diabet*" ) ) OR ( TITLE ( "non insulin dependent diabet*" ) ) OR ( TITLE ( "diabetes mellitus" ) ) OR ( ABS ( "diabetes mellitus" ) ) ) AND ( ( ABS ( "television viewing" ) ) OR ( TITLE ( "television viewing" ) ) OR ( ABS ( "screen time*" ) ) OR ( TITLE ( "screen time*" ) ) OR ( ABS ( "sitting" ) ) OR ( TITLE ( "sitting" ) ) OR ( ABS ( "sedentary time*" ) ) OR ( TITLE ( "sedentary time*" ) ) OR ( ABS ( "sedentary lifestyle*" ) ) OR ( TITLE ( "sedentary lifestyle*" ) ) OR ( ABS ( "sedentary behavior*" ) ) OR ( TITLE ( "sedentary behavior*" ) ) OR ( ABS ( "sedentary behaviour*" ) ) OR ( TITLE ( "sedentary behaviour*" ) ) ) AND PUBYEAR > 2021 AND PUBYEAR < 2025

**Web of Science, Sept, 16, 2022 = 2008**

1: TS=("Sedentary Behaviour*" ) Date run: Fri Sep 16 2022 14:32:38 GMT-0400 (Eastern Daylight Time) Results: 3820

2: TS=("Sedentary Behavior*") Date run: Fri Sep 16 2022 14:33:15 GMT-0400 (Eastern Daylight Time) Results: 11908

3: TS=("Sedentary Lifestyle*") Date run: Fri Sep 16 2022 14:33:37 GMT-0400 (Eastern Daylight Time) Results: 5793

4: TS=("sedentary time*") Date run: Fri Sep 16 2022 14:33:55 GMT-0400 (Eastern Daylight Time) Results: 4592

5: TS=("sitting") Date run: Fri Sep 16 2022 14:34:01 GMT-0400 (Eastern Daylight Time) Results: 32361

6: TS=("screen time*") Date run: Fri Sep 16 2022 14:34:10 GMT-0400 (Eastern Daylight Time) Results: 4854

7: TS=("television viewing") Date run: Fri Sep 16 2022 14:34:16 GMT-0400 (Eastern Daylight Time) Results: 2578

8: #7 OR #6 OR #5 OR #4 OR #3 OR #2 OR #1 Date run: Fri Sep 16 2022 14:34:28 GMT-0400 (Eastern Daylight Time) Results: 56361

9: TS=("diabetes mellitus") Date run: Fri Sep 16 2022 14:34:47 GMT-0400 (Eastern Daylight Time) Results: 287477

10: TS=("non insulin dependent diabet*") Date run: Fri Sep 16 2022 14:34:52 GMT-0400 (Eastern Daylight Time) Results: 9789

11: TS=("type 2 diabet*") Date run: Fri Sep 16 2022 14:35:31 GMT-0400 (Eastern Daylight Time) Results: 202144

12: #9 OR #10 OR #11 Date run: Fri Sep 16 2022 14:35:52 GMT-0400 (Eastern Daylight Time) Results: 404806

13: #8 AND #12 Date run: Fri Sep 16 2022 14:36:04 GMT-0400 (Eastern Daylight Time) Results: 2008

**Web of Science, Jan, 3, 2024 = 221**

1: TS=("Sedentary Behaviour*" ) Results: 4285

2: TS=("Sedentary Behavior*") Results: 13720

3: TS=("Sedentary Lifestyle*") Results: 6406

4: TS=("sedentary time*") Results: 5221

5: TS=("sitting") Results: 34623

6: TS=("screen time*") Results: 5972

7: TS=("television viewing") Results: 2629

8: #7 OR #6 OR #5 OR #4 OR #3 OR #2 OR #1 Results: 62062

9: TS=("diabetes mellitus") Results: 309345

10: TS=("non insulin dependent diabet*") Results: 9793

11: TS=("type 2 diabet*") Results: 221111

12: #9 OR #10 OR #11 Results: 437001

13: #8 AND #12 Timespan: 2022-09-16 to 2024-01-03 Results: 221

**PsycINFO, Sept 16, 2022 = 193**

1 exp Sedentary Behavior/ 2195

2 exp Screen Time/ 671

3 exp Television Viewing/ 4391

4 exp Posture/ 6746

5 "Sedentary Behaviour*".tw. 875

6 "Sedentary behavior*".tw. 2544

7 "Sedentary Lifestyle*".tw. 984

8 "Sedentary time*".tw. 925

9 1 or 2 or 3 or 4 or 5 or 6 or 7 or 8 16036

10 "diabetes mellitus".tw. 9075

11 "non insulin dependent diabet*".tw. 203

12 "type 2 diabet*".tw. 8368

13 exp Diabetes Mellitus/ 9745

14 exp Type 2 Diabetes/ 5464

15 10 or 11 or 12 or 13 or 14 16459

16 9 and 15 193

**PsycINFO, Jan 3, 2024 = 26**

1 exp Sedentary Behavior/ 2624

2 exp Screen Time/ 967

3 exp Television Viewing/ 4478

4 exp Posture/ 7175

5 "Sedentary Behaviour*".tw. 1035

6 "Sedentary behavior*".tw. 2871

7 "Sedentary Lifestyle*".tw. 1057

8 "Sedentary time*".tw. 1073

9 1 or 2 or 3 or 4 or 5 or 6 or 7 or 8 17406

10 "diabetes mellitus".tw. 9692

11 "non insulin dependent diabet*".tw. 205

12 "type 2 diabet*".tw. 9069

13 exp Diabetes Mellitus/ 10623

14 exp Type 2 Diabetes/ 6075

15 10 or 11 or 12 or 13 or 14 17666

16 9 and 15 214

16 limit 16 to yr=“2022-Current” 26

**Sport Discus, Sept, 16, 2022 = 25**

S16 S14 AND S15 Expanders - Apply equivalent subjects

Search modes - Boolean/Phrase Interface - EBSCOhost Research Databases

Search Screen - Advanced Search

Database - SPORTDiscus 25

S15 S11 OR S12 OR S13 Expanders - Apply equivalent subjects

Search modes - Boolean/Phrase Interface - EBSCOhost Research Databases

Search Screen - Advanced Search

Database - SPORTDiscus 1,895

S14 (S1 OR S2 OR S3 OR S4 OR S5 OR S6 OR S7 OR S8 OR S9 OR S10) Expanders - Apply equivalent subjects

Search modes - Boolean/Phrase Interface - EBSCOhost Research Databases

Search Screen - Advanced Search

Database - SPORTDiscus 4,788

S13 KW "type 2 diabet*" Expanders - Apply equivalent subjects

Search modes - Boolean/Phrase Interface - EBSCOhost Research Databases

Search Screen - Advanced Search

Database - SPORTDiscus 994

S12 KW "non insulin dependent diabet*" Expanders - Apply equivalent subjects

Search modes - Boolean/Phrase Interface - EBSCOhost Research Databases

Search Screen - Advanced Search

Database - SPORTDiscus 11

S11 KW "diabetes mellitus" Expanders - Apply equivalent subjects

Search modes - Boolean/Phrase Interface - EBSCOhost Research Databases

Search Screen - Advanced Search

Database - SPORTDiscus 1,207

S10 KW "television viewing” Expanders - Apply equivalent subjects

Search modes - Boolean/Phrase Interface - EBSCOhost Research Databases

Search Screen - Advanced Search

Database - SPORTDiscus 23

S9 KW "screen time*" Expanders - Apply equivalent subjects

Search modes - Boolean/Phrase Interface - EBSCOhost Research Databases

Search Screen - Advanced Search

Database - SPORTDiscus 155

S8 KW "sedentary time*" Expanders - Apply equivalent subjects

Search modes - Boolean/Phrase Interface - EBSCOhost Research Databases

Search Screen - Advanced Search

Database - SPORTDiscus 114

S7 KW "Sedentary Lifestyle*" Expanders - Apply equivalent subjects

Search modes - Boolean/Phrase Interface - EBSCOhost Research Databases

Search Screen - Advanced Search

Database - SPORTDiscus 231

S6 KW "Sedentary Behavior*" Expanders - Apply equivalent subjects

Search modes - Boolean/Phrase Interface - EBSCOhost Research Databases

Search Screen - Advanced Search

Database - SPORTDiscus 628

S5 KW "Sedentary Behaviour*" Expanders - Apply equivalent subjects

Search modes - Boolean/Phrase Interface - EBSCOhost Research Databases

Search Screen - Advanced Search

Database - SPORTDiscus 197

S4 DE "SITTING position" Expanders - Apply equivalent subjects

Search modes - Boolean/Phrase Interface - EBSCOhost Research Databases

Search Screen - Advanced Search

Database - SPORTDiscus 1,452

S3 DE "SEDENTARY people" Expanders - Apply equivalent subjects

Search modes - Boolean/Phrase Interface - EBSCOhost Research Databases

Search Screen - Advanced Search

Database - SPORTDiscus 139

S2 DE "SEDENTARY lifestyle" Expanders - Apply equivalent subjects

Search modes - SmartText Searching Interface - EBSCOhost Research Databases

Search Screen - Advanced Search

Database - SPORTDiscus 3,900

S1 DE "SEDENTARY behavior" Expanders - Apply equivalent subjects

Search modes - Boolean/Phrase Interface - EBSCOhost Research Databases

Search Screen - Advanced Search

Database - SPORTDiscus 1,279

­

**Sport Discus, Jan 3, 2024 = 1**

S16 S14 AND S15 Limiters - Publication Date: 20220901-20240231

Expanders - Apply equivalent subjects

Search modes - Boolean/Phrase View Results (1)

S15 S11 OR S12 OR S13 Expanders - Apply equivalent subjects

Search modes - Boolean/Phrase View Results (2,066)

S14 S1 OR S2 OR S3 OR S4 OR S5 OR S6 OR S7 OR S8 OR S9 OR S10 Expanders - Apply equivalent subjects

Search modes - Boolean/Phrase View Results (5,225)

S13 KW "type 2 diabet*" Expanders - Apply equivalent subjects

Search modes - Boolean/Phrase View Results (1,090)

S12 KW "non insulin dependent diabet*" Expanders - Apply equivalent subjects

Search modes - Boolean/Phrase View Results (11)

S11 KW "diabetes mellitus" Expanders - Apply equivalent subjects

Search modes - Boolean/Phrase View Results (1,321)

S10 KW "television viewing” Expanders - Apply equivalent subjects

Search modes - Boolean/Phrase View Results (23)

S9 KW "screen time*" Expanders - Apply equivalent subjects

Search modes - Boolean/Phrase View Results (191)

S8 KW "sedentary time*" Expanders - Apply equivalent subjects

Search modes - Boolean/Phrase View Results (130)

S7 KW "Sedentary Lifestyle*" Expanders - Apply equivalent subjects

Search modes - Boolean/Phrase View Results (254)

S6 KW "Sedentary Behavior*" Expanders - Apply equivalent subjects

Search modes - Boolean/Phrase View Results (764)

S5 KW "Sedentary Behaviour*" Expanders - Apply equivalent subjects

Search modes - Boolean/Phrase View Results (233)

S4 DE "SITTING position" Expanders - Apply equivalent subjects

Search modes - Boolean/Phrase View Results (1,547)

S3 DE "SEDENTARY people" Expanders - Apply equivalent subjects

Search modes - Boolean/Phrase View Results (142)

S2 DE "SEDENTARY lifestyle" Expanders - Apply equivalent subjects

Search modes - SmartText Searching View Results (4,258)

S1 DE "SEDENTARY behavior" Expanders - Apply equivalent subjects

Search modes - Boolean/Phrase View Results (1,469)

**CINAHL, Sept 16, 2022 = 1333**

S15 S9 AND S14 Expanders - Apply equivalent subjects

Search modes - Boolean/Phrase Interface - EBSCOhost Research Databases

Search Screen - Advanced Search

Database - CINAHL 1,333

S14 S10 OR S11 OR S12 OR S13 Expanders - Apply equivalent subjects

Search modes - Boolean/Phrase Interface - EBSCOhost Research Databases

Search Screen - Advanced Search

Database - CINAHL 197,571

S13 ""type 2 diabet*"" Expanders - Apply equivalent subjects

Search modes - Boolean/Phrase Interface - EBSCOhost Research Databases

Search Screen - Advanced Search

Database - CINAHL 88,558

S12 ""non insulin dependent diabet*"" Expanders - Apply equivalent subjects

Search modes - Boolean/Phrase Interface - EBSCOhost Research Databases

Search Screen - Advanced Search

Database - CINAHL 1,429

S11 ""diabetes mellitus"" Expanders - Apply equivalent subjects

Search modes - Boolean/Phrase Interface - EBSCOhost Research Databases

Search Screen - Advanced Search

Database - CINAHL 187,213

S10 MH "Diabetes Mellitus, Type 2" Expanders - Apply equivalent subjects

Search modes - Boolean/Phrase Interface - EBSCOhost Research Databases

Search Screen - Advanced Search

Database - CINAHL 69,446

S9 S1 OR S2 OR S3 OR S4 OR S5 OR S6 OR S7 OR S8 Expanders - Apply equivalent subjects

Search modes - Boolean/Phrase Interface - EBSCOhost Research Databases

Search Screen - Advanced Search

Database - CINAHL 26,928

S8 ""Sedentary time*"" Expanders - Apply equivalent subjects

Search modes - Boolean/Phrase Interface - EBSCOhost Research Databases

Search Screen - Advanced Search

Database - CINAHL 3,110

S7 ""Sedentary Behaviour*"" Expanders - Apply equivalent subjects

Search modes - Boolean/Phrase Interface - EBSCOhost Research Databases

Search Screen - Advanced Search

Database - CINAHL 1,791

S6 ""Sedentary Lifestyle*"" Expanders - Apply equivalent subjects

Search modes - Boolean/Phrase Interface - EBSCOhost Research Databases

Search Screen - Advanced Search

Database - CINAHL 6,346

S5 ""Sedentary behavior*"" Expanders - Apply equivalent subjects

Search modes - Boolean/Phrase Interface - EBSCOhost Research Databases

Search Screen - Advanced Search

Database - CINAHL 6,413

S4 MH "Television" Expanders - Apply equivalent subjects

Search modes - Boolean/Phrase Interface - EBSCOhost Research Databases

Search Screen - Advanced Search

Database - CINAHL 10,351

S3 MH "Screen Time" Expanders - Apply equivalent subjects

Search modes - Boolean/Phrase Interface - EBSCOhost Research Databases

Search Screen - Advanced Search

Database - CINAHL 845

S2 MH "Sitting" Expanders - Apply equivalent subjects

Search modes - Boolean/Phrase Interface - EBSCOhost Research Databases

Search Screen - Advanced Search

Database - CINAHL 3,208

S1 MH "Life Style, Sedentary+" Expanders - Apply equivalent subjects

Search modes - Boolean/Phrase Interface - EBSCOhost Research Databases

Search Screen - Advanced Search

Database - CINAHL 10,140

**CINAHL, Jan 3, 2024 = 124**

S15 S9 AND S14 Limiters – Publication Date: 20220901-20240231 Expanders - Apply equivalent subjects

Search modes - Boolean/Phrase Interface - EBSCOhost Research Databases

Search Screen - Advanced Search

Database - CINAHL 124

S14 S10 OR S11 OR S12 OR S13 Expanders - Apply equivalent subjects

Search modes - Boolean/Phrase Interface - EBSCOhost Research Databases

Search Screen - Advanced Search

Database - CINAHL 203,156

S13 ""type 2 diabet*"" Expanders - Apply equivalent subjects

Search modes - Boolean/Phrase Interface - EBSCOhost Research Databases

Search Screen - Advanced Search

Database - CINAHL 64,195

S12 ""non insulin dependent diabet*"" Expanders - Apply equivalent subjects

Search modes - Boolean/Phrase Interface - EBSCOhost Research Databases

Search Screen - Advanced Search

Database - CINAHL 1,299

S11 ""diabetes mellitus"" Expanders - Apply equivalent subjects

Search modes - Boolean/Phrase Interface - EBSCOhost Research Databases

Search Screen - Advanced Search

Database - CINAHL 191,916

S10 MH "Diabetes Mellitus, Type 2" Expanders - Apply equivalent subjects

Search modes - Boolean/Phrase Interface - EBSCOhost Research Databases

Search Screen - Advanced Search

Database - CINAHL 70,739

S9 S1 OR S2 OR S3 OR S4 OR S5 OR S6 OR S7 OR S8 Expanders - Apply equivalent subjects

Search modes - Boolean/Phrase Interface - EBSCOhost Research Databases

Search Screen - Advanced Search

Database - CINAHL 27,832

S8 ""Sedentary time*"" Expanders - Apply equivalent subjects

Search modes - Boolean/Phrase Interface - EBSCOhost Research Databases

Search Screen - Advanced Search

Database - CINAHL 2,078

S7 ""Sedentary Behaviour*"" Expanders - Apply equivalent subjects

Search modes - Boolean/Phrase Interface - EBSCOhost Research Databases

Search Screen - Advanced Search

Database - CINAHL 6,024

S6 ""Sedentary Lifestyle*"" Expanders - Apply equivalent subjects

Search modes - Boolean/Phrase Interface - EBSCOhost Research Databases

Search Screen - Advanced Search

Database - CINAHL 6,766

S5 ""Sedentary behavior*"" Expanders - Apply equivalent subjects

Search modes - Boolean/Phrase Interface - EBSCOhost Research Databases

Search Screen - Advanced Search

Database - CINAHL 6,913

S4 MH "Television" Expanders - Apply equivalent subjects

Search modes - Boolean/Phrase Interface - EBSCOhost Research Databases

Search Screen - Advanced Search

Database - CINAHL 10,140

S3 MH "Screen Time" Expanders - Apply equivalent subjects

Search modes - Boolean/Phrase Interface - EBSCOhost Research Databases

Search Screen - Advanced Search

Database - CINAHL 1,253

S2 MH "Sitting" Expanders - Apply equivalent subjects

Search modes - Boolean/Phrase Interface - EBSCOhost Research Databases

Search Screen - Advanced Search

Database - CINAHL 3,290

S1 MH "Life Style, Sedentary+" Expanders - Apply equivalent subjects

Search modes - Boolean/Phrase Interface - EBSCOhost Research Databases

Search Screen - Advanced Search

Database - CINAHL 11,403

**Cochrane Library, Sept 16, 2022 = 459 (trials 453/ reviews 6)**

#1 MeSH descriptor: [Diabetes Mellitus, Type 2] explode all trees 20214

#2 ("type 2 diabet*"):ti,ab,kw (Word variations have been searched) 40140

#3 ("non insulin dependent diabet*"):ti,ab,kw 1

#4 ("diabetes mellitus"):ti,ab,kw 73069

#5 #1 OR #2 OR #3 OR #4 78851

#6 MeSH descriptor: [Sedentary Behavior] explode all trees 1350

#7 MeSH descriptor: [Sitting Position] explode all trees 152

#8 MeSH descriptor: [Screen Time] explode all trees 33

#9 MeSH descriptor: [Television] explode all trees 1565

#10 ("Sedentary Behaviour*"):ti,ab,kw 2608

#11 ("Sedentary Behavior*"):ti,ab,kw 2608

#12 ("Sedentary Lifestyle*"):ti,ab,kw 1771

#13 ("Sedentary time*"):ti,ab,kw 957

#14 #6 OR #7 OR #8 OR #9 OR #10 OR #11 OR #12 OR #13 5833

#15 #5 AND #14 459

**Cochrane Library, Jan 1, 2024 = 88 (trials 88)**

#1 MeSH descriptor: [Diabetes Mellitus, Type 2] explode all trees 23599

#2 ("type 2 diabet*"):ti,ab,kw (Word variations have been searched) 61071

#3 ("non insulin dependent diabet*"):ti,ab,kw 23102

#4 ("diabetes mellitus"):ti,ab,kw 82050

#5 #1 OR #2 OR #3 OR #4 90924

#6 MeSH descriptor: [Sedentary Behavior] explode all trees 1609

#7 MeSH descriptor: [Sitting Position] explode all trees 222

#8 MeSH descriptor: [Screen Time] explode all trees 68

#9 MeSH descriptor: [Television] explode all trees 1686

#10 ("Sedentary Behaviour*"):ti,ab,kw 1389

#11 ("Sedentary Behavior*"):ti,ab,kw 3795

#12 ("Sedentary Lifestyle*"):ti,ab,kw 3284

#13 ("Sedentary time*"):ti,ab,kw 5213

#14 #6 OR #7 OR #8 OR #9 OR #10 OR #11 OR #12 OR #13 9573

#15 #5 AND #14 88

with Cochrane Library publication date from Sep 2022 to Feb 2024
