# Supplementary material for: Interventions to reduce sedentary behaviour in adults with type 2 diabetes: A systematic review and meta-analysis
Source: PLoS One. 2024 Jul 30;19(7):e0306439. doi: 10.1371/journal.pone.0306439 (PMC11288443; doi:10.1371/journal.pone.0306439)
Supplement: Attachment — Submitted filename: Response to Reviewers.docx [file pone.0306439.s005.docx]

**Journal requirements:**

When submitting your revision, we need you to address these additional requirements.

1. Please ensure that your manuscript meets PLOS ONE's style requirements, including those for file naming. The PLOS ONE style templates can be found at
<https://journals.plos.org/plosone/s/file?id=wjVg/PLOSOne_formatting_sample_main_body.pdf> and
<https://journals.plos.org/plosone/s/file?id=ba62/PLOSOne_formatting_sample_title_authors_affiliations.pdf>

**Response:**

We have tried to adhere to PLOS ONE’s style requirements. Including the title page, abstract, body, headings, figures (formatting, citations, and captions), tables (formatting, citations, and captions), file naming, and referencing.

2. We notice that your supplementary figures are uploaded with the file type 'Figure'. Please amend the file type to 'Supporting Information'. Please ensure that each Supporting Information file has a legend listed in the manuscript after the references list.

**Response:**

We have added the supplementary information in the manuscript after the reference list:

S1 Table. Short-term SB interventions

S2 Table. Longer-term SB interventions

S3 Table. Quality of eligible interventions

S1 File. Database searches

We have also amended the file types to Supporting Information.

**Comments to the Author:**

1. Is the manuscript technically sound, and do the data support the conclusions?

The manuscript must describe a technically sound piece of scientific research with data that supports the conclusions. Experiments must have been conducted rigorously, with appropriate controls, replication, and sample sizes. The conclusions must be drawn appropriately based on the data presented.

Reviewer #1: Partly

Reviewer #2: Yes

**Response:**

We aimed to provide a technically sound piece of scientific research with data that supports the conclusions. PRISMA guidelines were followed, a broad search was conducted with guidance from a university librarian, quality was assessed using the Cochrane Risk of Bias tools, and the review was pre-registered. Additionally, a meta-analysis of short-term SB interventions was conducted on glucose control, a meta-regression was conducted, and heterogeneity and publication bias were assessed.

2. Has the statistical analysis been performed appropriately and rigorously?

Reviewer #1: No

Reviewer #2: Yes

Response:

Our methods including our search strategy, inclusion/ exclusion criteria, selection of studies, data extraction and synthesis, quality assessment, and synthesis of results including meta-analysis are described in detail.

3. Have the authors made all data underlying the findings in their manuscript fully available?

The [PLOS Data policy](http://www.plosone.org/static/policies.action#sharing) requires authors to make all data underlying the findings described in their manuscript fully available without restriction, with rare exception (please refer to the Data Availability Statement in the manuscript PDF file). The data should be provided as part of the manuscript or its supporting information, or deposited to a public repository. For example, in addition to summary statistics, the data points behind means, medians and variance measures should be available. If there are restrictions on publicly sharing data—e.g. participant privacy or use of data from a third party—those must be specified.

Reviewer #1: Yes

Reviewer #2: Yes

**Response:**

Transparency is achieved by providing all methodology and data underlying the findings in the manuscript.

4. Is the manuscript presented in an intelligible fashion and written in standard English?

PLOS ONE does not copyedit accepted manuscripts, so the language in submitted articles must be clear, correct, and unambiguous. Any typographical or grammatical errors should be corrected at revision, so please note any specific errors here.

Reviewer #1: No

Reviewer #2: Yes

**Response:**

We have re-read the manuscript in detail to correct any remaining typographical and grammatical errors. Less clear and concise sentences have been modified accordingly.

5. Review Comments to the Author

Please use the space provided to explain your answers to the questions above. You may also include additional comments for the author, including concerns about dual publication, research ethics, or publication ethics. (Please upload your review as an attachment if it exceeds 20,000 characters).

Reviewer #1: Good effort was placed in this manuscript but lacks so many crucial elements to make an attractive read and source of information. It is very long with many repetitive paragraphs and ideas.

**Response:**

We have re-read the manuscript in detail to remove any repetitive paragraphs and ideas. For instance, we have removed a paragraph on page 5, we have condensed six paragraphs into two on page 18 and 19, we have removed two repetitive ideas on page 23. Further, throughout the manuscript we have tried to re-word sentences to be more concise.

The tables are huge with extensive information at makes not an optimal source of informative read. Summarizing information done for systemic review is crucial to produce a publication for readers.

**Response:**

We believe that our tables convey summarized vital information about each included studies objectives, participant characteristics, research design, intervention and comparison, sedentary behaviour outcomes, cardiometabolic outcomes, and results. We believe that summarizing them further would remove important information. However, we understand that the tables are quite long. Therefore, we have changed Table 1 and 3 to supplementary tables (S1 and S2). This will allow for an easier read of the manuscript while still allowing readers to access all the information provided in the supplementary tables. We also added a participant column and risk of bias column to Tables 2 and 4 (now Table 1 and 2) to provide more detail to the in-text tables.

Many sentences also were not coherent and understood. Many subtitles of paragraphs make the article less smooth to read. I recommend a total re-wright of the manuscript.

**Response:**

We have re-read the manuscript in detail to correct incoherent sentences. We have also removed six subtitles to allow the article to read easier including: “Description of studies”, “24-hour continuous interstitial glucose measures: intervention (breaks from sitting) vs control (sitting)”, “combined interstitial continuous postprandial glucose measures: intervention (breaks from sitting) vs control (sitting)”, “Qualitative synthesis of non-glucose outcomes”, “Qualitative synthesis” and “Main findings”

Reviewer #2: Thank you for offering me an invitation to be the reviewer of this manuscript” Interventions to reduce sedentary behaviour in adults with type 2 diabetes: A systematic review and meta-analysis” Siobhan et al had submitted a Systematic review and meta – analysis of literature on interventions to reduce SB in T2DM. I am giving my observations and comments for the author to consider:
The flow of the manuscript is very comfortable. The language used is very professional and easy to follow. Text formatting may be looked into.

**Response:**

We have now formatted the manuscript to PLOS ONE's style requirements.

This systematic review and meta – analysis is observed to have followed rigorous methodology, have applied relevant search strategies and analysis methods. The types of studies included in this systematic review are thoroughly described. The PRISMA flow diagram is easy to follow and complete.

**Response:**

Thank you, Reviewer 2.

Page 22 under Qualitative synthesis of non glucose outcomes: it should be “Table 2".

**Response:**

This has been fixed.

The discussion is detailed and elaborate. The metanalysis findings of short-term interventions and the narratives of non-glucose outcomes and long-term interventions are well discussed through. All the limitations of this review are mentioned.

**Response:**

Thank you, Reviewer 2.

I recommend this article may be published with the suggested minor corrections.

**Response:**

Thank you, Reviewer 2.

6. PLOS authors have the option to publish the peer review history of their article ([what does this mean?](https://journals.plos.org/plosone/s/editorial-and-peer-review-process#loc-peer-review-history)). If published, this will include your full peer review and any attached files. If you choose “no”, your identity will remain anonymous but your review may still be made public. Do you want your identity to be public for this peer review? For information about this choice, including consent withdrawal, please see our [Privacy Policy](https://www.plos.org/privacy-policy).

Reviewer #1: No

Reviewer #2: No

**Response:**

Thank you, reviewers.

[NOTE: If reviewer comments were submitted as an attachment file, they will be attached to this email and accessible via the submission site. Please log into your account, locate the manuscript record, and check for the action link "View Attachments". If this link does not appear, there are no attachment files.]

**Response:**

There were no attached files.

While revising your submission, please upload your figure files to the Preflight Analysis and Conversion Engine (PACE) digital diagnostic tool, <https://pacev2.apexcovantage.com/>. PACE helps ensure that figures meet PLOS requirements. To use PACE, you must first register as a user. Registration is free. Then, login and navigate to the UPLOAD tab, where you will find detailed instructions on how to use the tool. If you encounter any issues or have any questions when using PACE, please email PLOS at [figures@plos.org](mailto:figures@plos.org). Please note that Supporting Information files do not need this step.

**Response:**

We have followed the above instructions.

**Closing Comments:**

As you can see from our resubmission and from the reviewers’ comments, we have conducted a systematic review and meta-analysis that provides important information for this field of study. One reviewer did not have any concerns with how the manuscript was put together. The other reviewer had concerns with the style of the manuscript. We have tried to address all these concerns by removing repetitive paragraphs and ideas, modifying less clear and concise sentences, moving larger tables to the supplementary information, and removing numerous subheadings. All together we believe it has resulted in a higher quality manuscript for your journal. We trust you feel the same way and look forward to your final editorial decision.
